# *GhSOS1*, a plasma membrane Na^+^/H^+^ antiporter gene from upland cotton, enhances salt tolerance in transgenic *Arabidopsis thaliana*

**DOI:** 10.1371/journal.pone.0181450

**Published:** 2017-07-19

**Authors:** Xiugui Chen, Xuke Lu, Na Shu, Delong Wang, Shuai Wang, Junjuan Wang, Lixue Guo, Xiaoning Guo, Weili Fan, Zhongxu Lin, Wuwei Ye

**Affiliations:** 1 State Key Laboratory of Cotton Biology/Institute of Cotton Research of Chinese Academy of Agricultural Sciences, Anyang, Henan, China; 2 National Key Laboratory of Crop Genetic Improvement, College of Plant Sciences and Technology, Huazhong Agricultural University, Wuhan, Hubei, China; National Taiwan University, TAIWAN

## Abstract

Upland cotton (*Gossypium hirsutum* L.), an important source of natural fiber, can tolerate relatively high salinity and drought stresses. In the present study, a plasma membrane Na^+^/H^+^ antiporter gene, *GhSOS1*, was cloned from a salt-tolerant genotype of *G*. *hirsutum*, Zhong 9807. The expression level of *GhSOS1* in cotton roots was significantly upregulated in the presence of high concentrations of NaCl (200 mM), while its transcript abundance was increased when exposed to low temperature and drought stresses. Localization analysis using onion epidermal cells showed that the GhSOS1 protein was localized to the plasma membrane. The overexpression of *GhSOS1* in *Arabidopsis* enhanced tolerance to salt stress, as indicated by a lower MDA content and decreased Na^+^/K^+^ ratio in transgenic plants. Moreover, the transcript levels of stress-related genes were significantly higher in *GhSOS1* overexpression lines than in wild-type plants under salt treatment. Hence, *GhSOS1* may be a potential target gene for enhancing salt tolerance in transgenic plants.

## Introduction

Soil salinity is one of the major threats to agricultural productivity because it disturbs intracellular ion homeostasis and reduces metabolic activities in plants [1]. Excess sodium ions (Na^+^) lead to water deficiency, membrane dysfunction, and ionic toxicity in plant cells [2]. Thus, it is essential for plants to maintain a low level of Na^+^ in the cytosol under salt stress. Plants have three mechanisms to prevent excessive Na^+^ accumulation in the cytosol: restricting influx, increasing efflux, and increasing Na^+^ sequestration into vacuoles [3]. Maintaining a low level of Na^+^ in the cytosol is largely mediated through transporters localized on the plasma membrane and tonoplasts [4].

In *Arabidopsis*, the plasma membrane Na^+^/H^+^ antiporter gene *AtSOS1* was identified as one component of the Salt Overly Sensitive (SOS) signal transduction pathway [5]. The SOS pathway comprises three components, SOS1, SOS2, and SOS3, which play important roles in maintaining ion homeostasis and controlling salt tolerance in plants [6]. SOS1 plays a critical role in Na^+^ extrusion and controlling the long-distance transport of Na^+^ from root to shoot[5]. SOS2 is a serine/threonine protein kinase [7] that interacts with and is activated by SOS3 [8]. SOS3, as a myristoylated EF-hand calcium-binding protein, senses and interprets the cellular calcium signal elicited under salt stress [9]. The SOS2/SOS3 protein kinase complex phosphorylates and activates SOS1 [10]. Recently, a series of *SOS1* homologs have been identified and cloned from a number of other plant species, such as *Populus euphratica* [11], rice (*Oryza sativa*) [12], wheat (*Triticum aestivum*) [13], tomato (*Lycopersicon esculentum*) [14], *Thellungiella salsuginea* [15], *Physcomitrella patens* [16], *Chrysanthemum crissum* [17], and *Helianthus tuberosus* [18]. SOS1 can partially suppress the salt-sensitive phenotype of yeasts without Na^+^ efflux transporters[13–18] and transport Na^+^ out of plant cells. The *SlSOS1*-silenced transgenic tomato plants accumulated more Na^+^ in the leaves and roots [14], but *HtSOS1* overexpression in rice could exclude more Na^+^ and accumulate more K^+^ [18]. The sos1 mutant lines of *Thellungiella salsuginea* [15] and *Physcomitrella patens* [16] showed excessive Na^+^ accumulation in cells.

Upland cotton (*Gossypium hirsutum* L.) is an economically important crop that provides natural fiber and foodstuffs worldwide. As a glycophytic plant, cotton shows higher salt and drought tolerance than other major crops[19, 20]. However, the limitation of water and salinization of cotton cultivation areas are a challenge for cotton production. Studies on the salt tolerance genes of cotton will benefit improvements in productivity under saline conditions and have great economic value. However, information on the cotton SOS pathway is limited. With the recent availability of upland cotton reference genome sequences [21], the functional study of cotton genes has become very effective and convenient.

In the present study, we isolated and characterized the plasma membrane Na^+^/H^+^ antiporter gene *GhSOS1* in upland cotton and demonstrated that the encoded protein was localized to the plasma membrane and its expression was upregulated under salt, drought and cold treatments. Furthermore, *GhSOS1* overexpression enhanced tolerance to high salt stress in transgenic *Arabidopsis* through maintaining a low Na^+^/K^+^ ratio and activating salt stress-related genes in cells. Hence, *GhSOS1* may be a target gene for enhancing the salt tolerance of transgenic plants.

## Material and methods

### Plant materials and stress treatments

Seeds of upland cotton Zhong 9807 were obtained from the Institute of Cotton Research of Chinese Academy of Agricultural Sciences and were planted in small pots with sand in a greenhouse with a 16-h light/8-h dark cycle at 28°C. To provide samples for gene expression analysis induced under abiotic stresses, plants at the three-leaf stage were moved to a liquid culture containing either 200 mM NaCl or 12% PEG 6000 (w/v), or maintained at 4°C. Each treatment was replicated three times. The roots and leaves under the various treatments mentioned above were harvested at 0, 1, 3, 6, 12 and 24 h, frozen in liquid nitrogen and stored at -80°C until use for RNA extraction.

### Cloning and bioinformatic analysis of *GhSOS1*

According to the protein sequence encoded by the *AtSOS1* gene (GenBank accession AAF76139.1), the *G*. *hirsutum* gene, named CotAD_24605, was detected in the cotton genome database (http://cgp.genomics.org.cn). Total RNA was extracted from cotton seedling leaves using the TRIzol reagent (Aidlab Biotech, Beijing, China) according to the manufacturer’s instructions. A pair of primers (S1 Table) targeting *GhSOS1* was designed based on its open reading frame (ORF). Full-length *GhSOS1* was obtained through PCR using this pair primer. The resulting amplicon was purified using the Agarose Gel DNA Purification Kit (Transgen Biotech, Beijing, China), ligated into the pMD19-T vector (TaKaRa BIOTECH, Dalian, China) and confirmed through DNA sequencing. MEGA software (version 5.10) [22]was used to perform multiple peptide alignments and phylogenetic analyses of the *GhSOS1* protein.

### Subcellular localization of GhSOS1

The ORF of *GhSOS1* without the stop codon was PCR amplified using specific primers (S1 Table). The PCR products were subsequently fused to the N-terminus of the GFP expression vector under the control of the 35S promoter. Plasmid DNA with 35S::*GhSOS1*-*GFP* and 35S::*GFP* was subsequently transformed into onion epidermal cells using the particle bombardment method. Transformed onion epidermal cells were cultured on MS media in the dark for 20 h at 25°C. The expression of the genes transformed into the onion epidermal cells was observed using a confocal laser scanning microscope (BX53F OLYMPUS, Tokyo, Japan).

### RNA isolation and quantitative real-time PCR

To determine the expression levels of *GhSOS1* in cotton seedlings treated with various abiotic stresses and analyze the expression of *GhSOS1* and the salt-related genes in the transgenic *Arabidopsis* plants, total RNA from cotton seedlings and *Arabidopsis* plants was respectively isolated from the collected tissues using TRIzol reagent (Aidlab Biotech). The amplification of quantitative real-time PCR products was performed in a reaction mixture of 20 μL of SYBR Green Master Mix (Transgen Biotech) according to the manufacturer’s instructions. Three biological replicates and three technical replicates for each sample were performed. The primers used for quantitative real-time PCR are shown in S1 Table.

### Yeast strains and media

The *Saccharomyces cerevisiae* strain AXT3 was used in the complementary assay, which has been described elsewhere [23]. The ORF of *GhSOS1* was amplified using gene-specific primers (S1 Table) and ligated into the pYES2 expression vector. The plasmid pYES2 was used as a negative control. Yeast transformation with the different plasmid constructs was performed using a standard lithium-PEG method. The cells were grown at 30°C in YPD medium (1% yeast extract, 2% peptone, and 2% glucose) or SC-U selective medium (0.67% yeast nitrogen base, 2% galactose, and 0.192% amino acid mixture without uracil), which was essentially free of alkali cations. Na^+^ tolerance in drop tests was performed in SC-U medium supplemented with different concentrations of NaCl (0, 50, 100 and 150 mM) as indicated, and grown for 2–4 days at 30°C[24].

### Construction of the *GhSOS1* overexpression vector and *Arabidopsis* transformation

The open reading frame of *GhSOS1* was cloned into the pBI121 vector and subsequently the vector was transferred into *Agrobacterium tumefaciens* strain LBA4404. *GhSOS1* transgenic *Arabidopsis* plants were generated through *Agrobacterium*-mediated transformation using the floral dipping method [25]. T_1_ and T_2_ seeds were screened on 50 mg/L kanamycin plates to select for the generation of homozygous progenies. Two randomly selected independent homozygous T_3_ lines were used for subsequent salt stress tolerance tests.

### Salt stress tolerance assay of *GhSOS1* overexpression in transgenic *Arabidopsis*

To assess salt tolerance of full grown *Arabidopsis* plants, wild-type and transgenic seeds were grown on MS plates for 10 days and the seedlings were subsequently transplanted into plastic containers filled with humus soil. After six weeks, wild-type and transgenic plants with similar growth states were selected and watered with the same volume of water or water containing 250 mM NaCl continuously for 7 days. The status of these plants was observed and photos were taken.

To determine the MDA content in plants under salt stress conditions, the leaves were collected from plants after water or NaCl treatment. The MDA content analysis was assessed according to the thiobarbituric acid method [26].

To measure the Na^+^ and K^+^ content, the leaves of wild-type and transgenic lines grown under normal conditions or 100 mM NaCl for 3 weeks were harvested. The followed steps were performed according to Rus *et al*. [27].

## Results

### Isolation and identification of the *GhSOS1* gene

**Fig 1:**
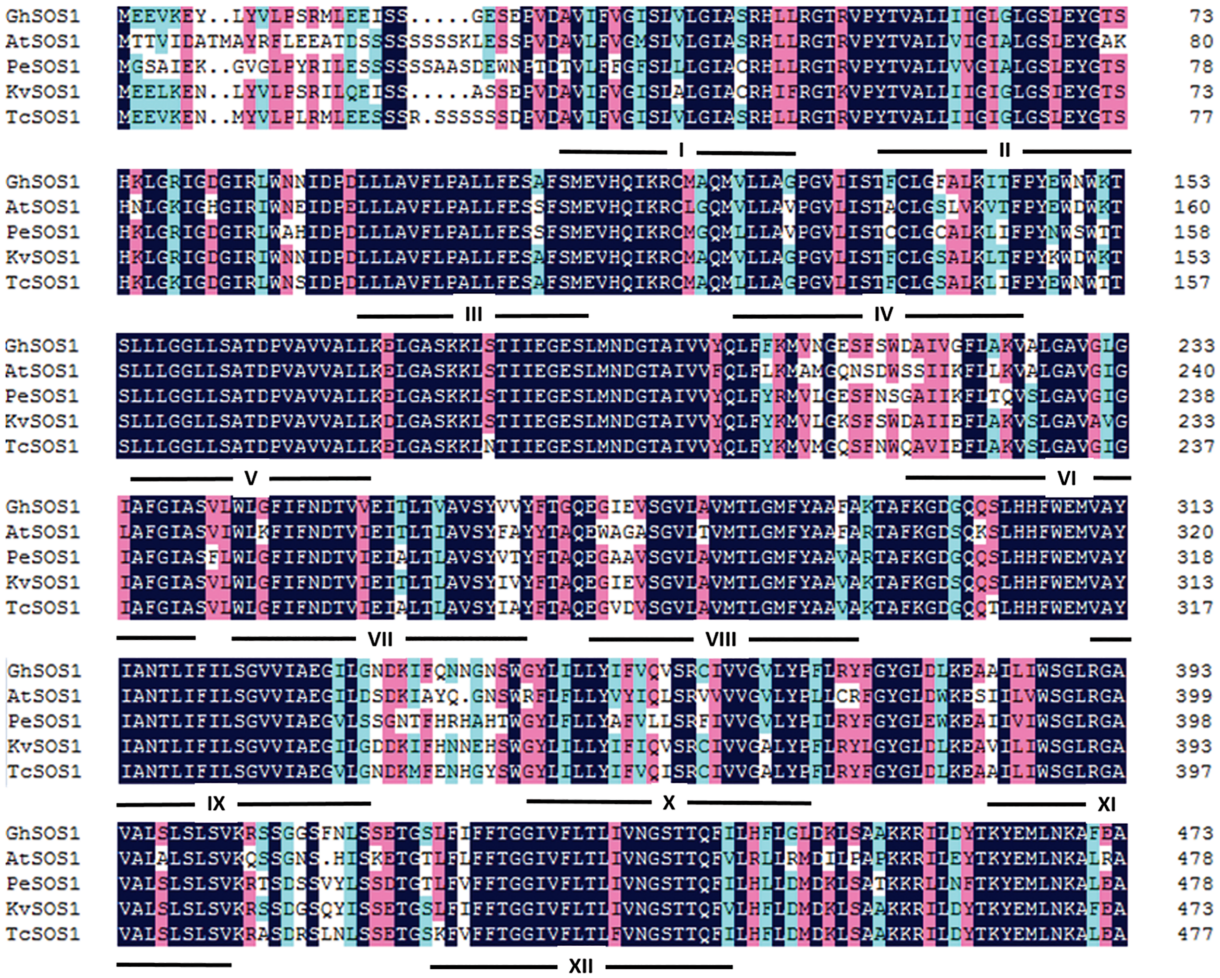
Alignment of GhSOS1 and SOS1 homologs of other plant species. *Arabidopsis thaliana* (AtSOS1, AF256224), *Populus euphratica* (PeSOS1, DQ517530), *Kosteletzkya virginica* (KvSOS1, KJ577576) and *Theobroma cacao* (TcSOS1, XM_007045345). Identical peptides are highlighted in black, and conservative substitutions are indicated in pink. Putative GhSOS1 transmembrane domains are underlined.

**Fig 2:**
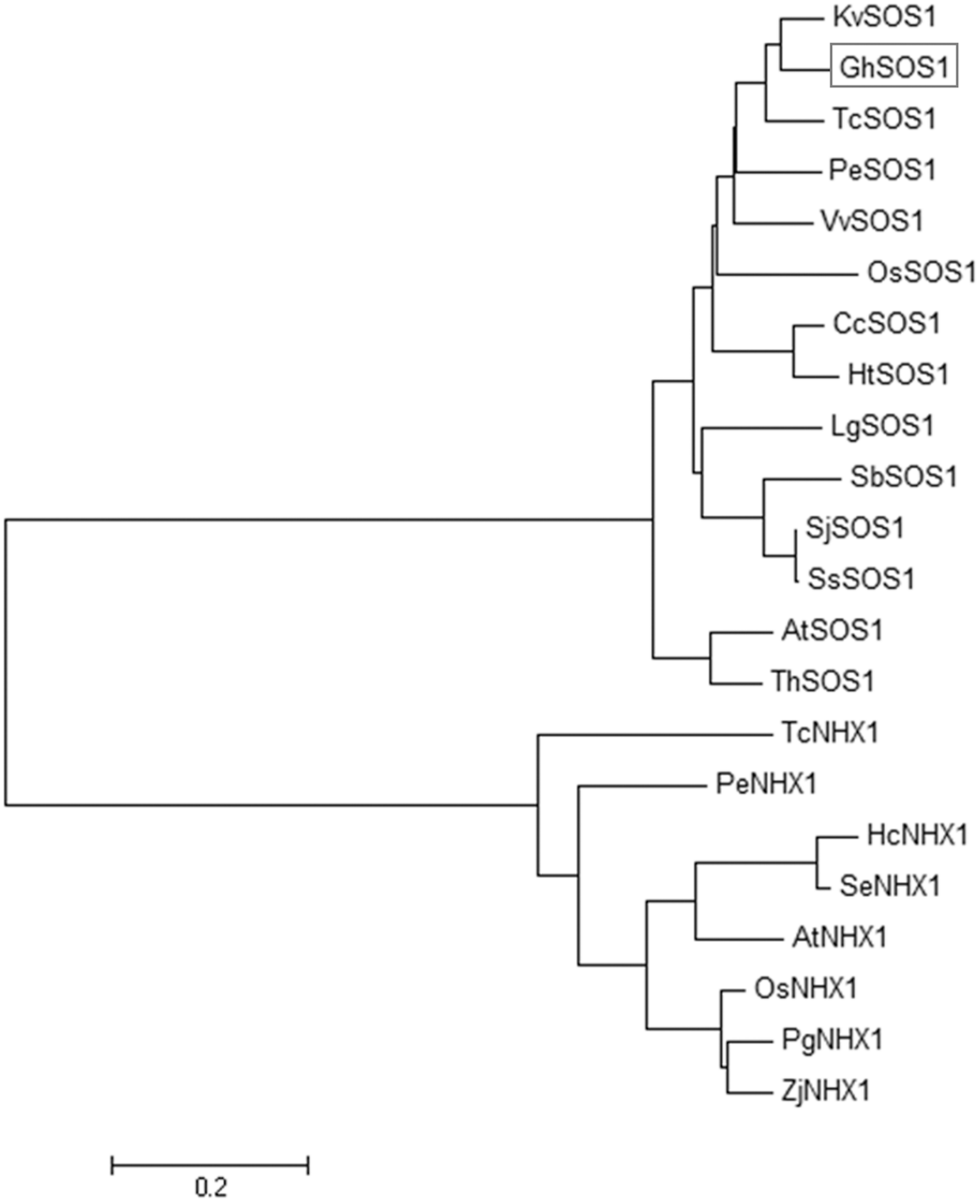
The phylogenetic relationship between GhSOS1 and SOS1 from other plant species. The phylogenetic tree was constructed using MEGA ver. 5.0. The following protein sequences were used to construct of the phylogenetic tree: *Arabidopsis thaliana* AtSOS1 (AF256224) and AtNHX1 (AF510074), *Chrysanthemum crassum* CcSOS1 (AB439132), *Halostachys caspica* HcNHX1 (GU188850), *Helianthus tuberosus* HtSOS1 (KC410809), *Kosteletzkya virginica* KvSOS1 (KJ577576), *Limonium gmelinii* LgSOS1 (EU780458), *Oryza sativa* OsSOS1 (AY785147) and OsNHX1 (AB021878), *Populus euphratica* PeSOS1 (DQ517530) and PeNHX1 (FJ866610), *Pennisetum glaucum* PgNHX1 (DQ071264), *Salicornia brachiata* SbSOS1 (EU879059), *Salicornia europaea* SeNHX1 (AY131235), *Suaeda japonica* SjSOS1 (AB198179), *Suaeda salsa* SsSOS1 (KF914414), *Theobroma cacao* TcSOS1 (XM_007045345) and TcNHX1 (XM_007030729), *Thellungiella halophila* ThSOS1 (EF207775), *Vitis vinifera* VvSOS1 (CAO42437) and *Zoysia japonica* ZjNHX1 (EU333827).

The ORF of *GhSOS1* was obtained using RT-PCR according to the sequence of CotAD_24605. The ORF of this gene was 3459 bp, and the predicted translation product was 1152 amino acids with a theoretical molecular mass of 128-kDa. The putative amino acid sequence shared greater similarity with those of the plasma membrane Na^+^/H^+^ antiporters isolated from plants (64–86%) (Fig 1). The phylogenetic analysis suggested that the sequence clustered closely with other plant plasma membrane Na^+^/H^+^ antiporters, and is most closely related to the *Kosteletzkya virginica* homolog (GenBank accession KJ577576.1). Its relationship with vacuolar Na^+^/H^+^ antiporters is not close (Fig 2).

### GhSOS1 was localized to the plasma membrane

**Fig 3:**
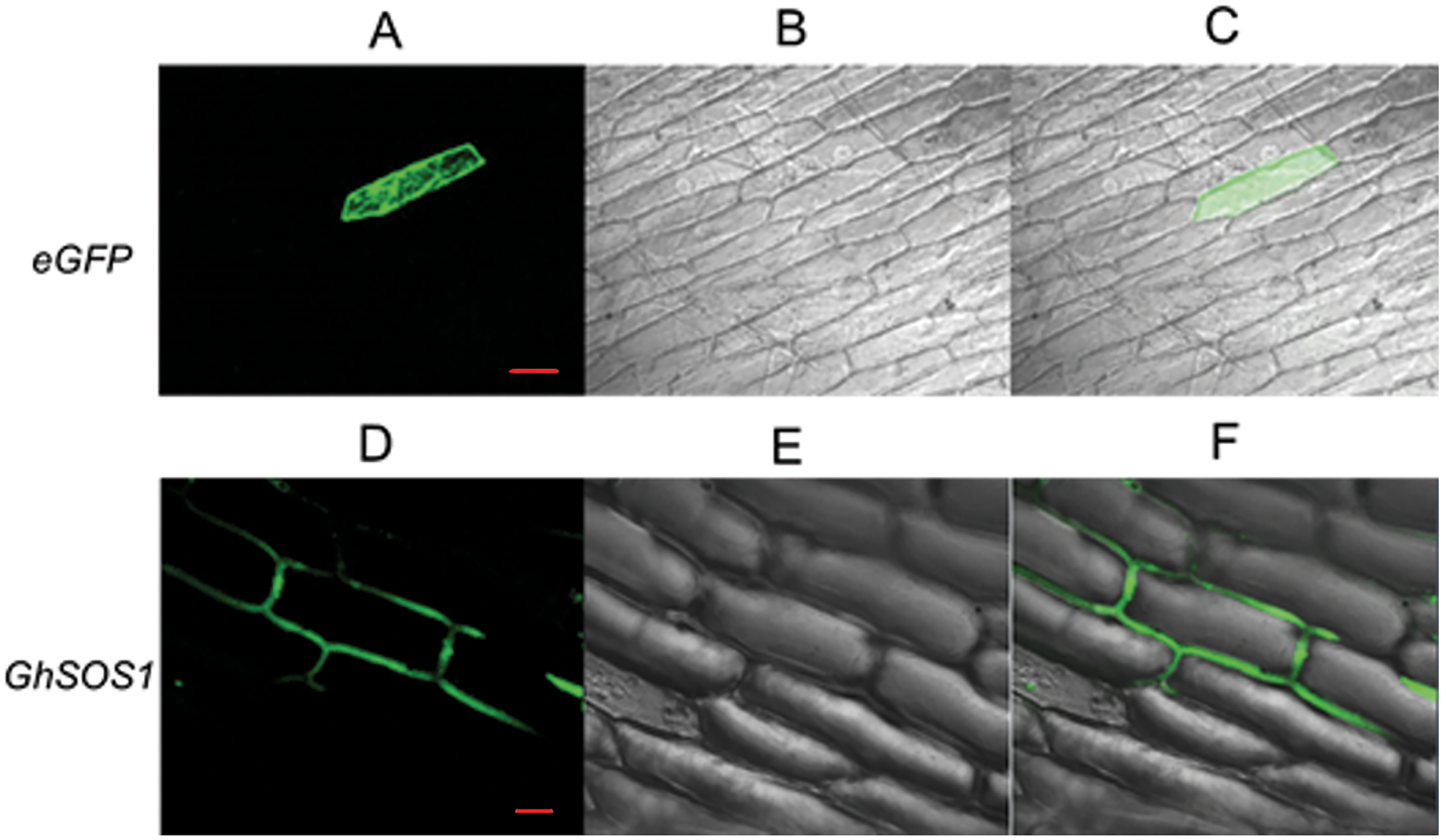
Localization of GhSOS1 in onion epidermal cells. A-C: Onion epidermal cells transformed with 35S::*GFP*. Bar: 100 μm. D-F: Onion epidermal cells transformed with 35S::*GFP*-*GhSOS1*. Bar: 50 μm. A and D: Dark field images for the detection of GFP fluorescence. B and E: Light field microscopy images to display morphology. C and F: Superimposed light and dark field images.

To investigate the subcellular localization of GhSOS1, we fused the GhSOS1 gene in frame with GFP and transiently expressed this gene in onion epidermal cells. Analyses of the transformed onion epidermal cells revealed that *GhSOS1* was only expressed near the plasma membrane (Fig 3), confirming that the *GhSOS1* gene encods a plasma membrane Na^+^/H^+^ antiporter.

### Transcriptional expression of *GhSOS1* in response to abiotic stresses

**Fig 4:**
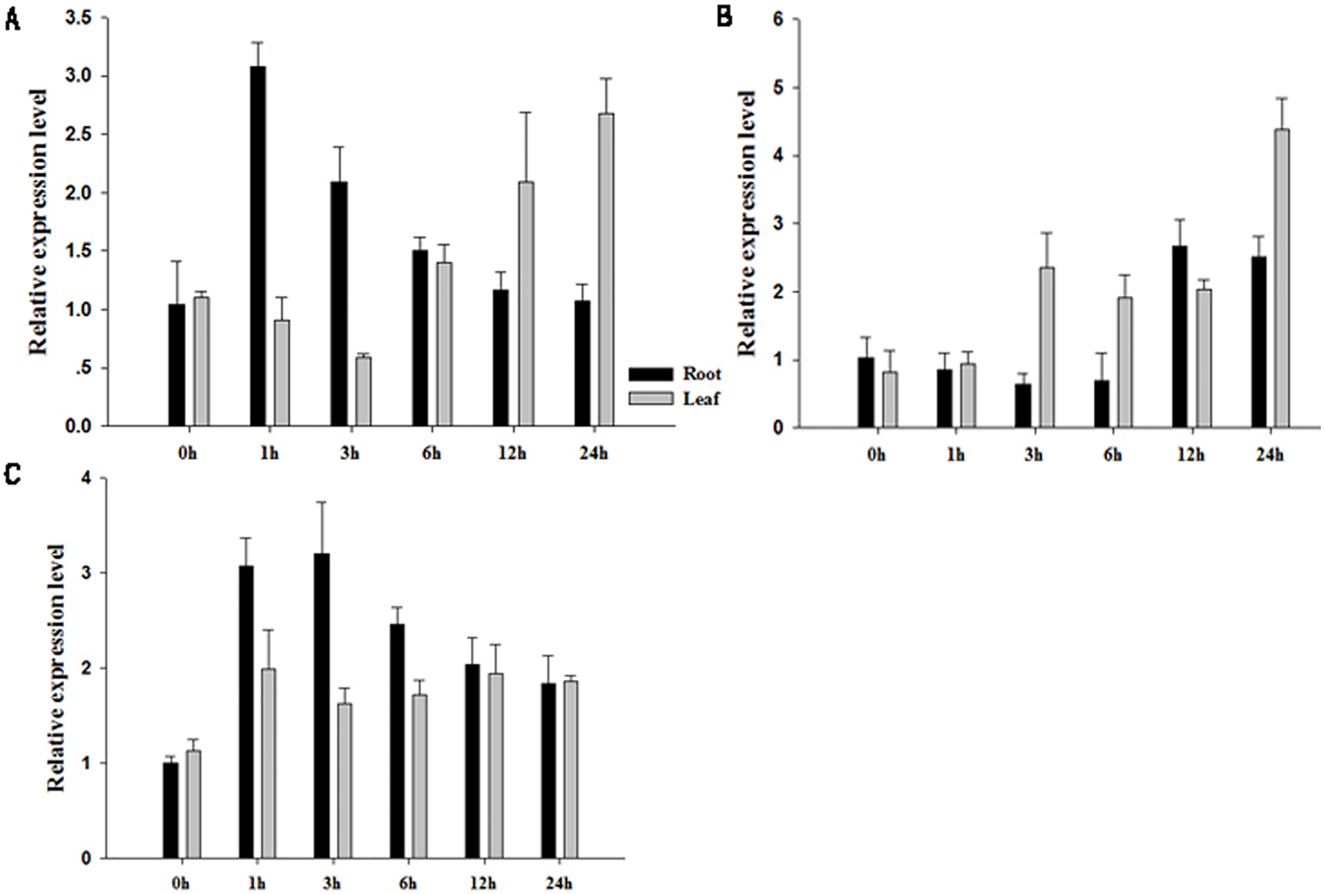
Expression analysis of *GhSOS1* in *G*. *hirsutum*. Plants exposed to (A) 200 mM NaCl, (B) 12% PEG 6000, and (C) low temperature (4°C).

Total RNA was isolated from cotton to investigate the expression patterns of *GhSOS1* under abiotic stresses through quantitative real-time PCR. Under salt stress, the expression level of *GhSOS1* in the roots initially increased approximately 3-fold at 1 h after treatment, while in the leaves, *GhSOS1* expression increased after 6 h and reached approximately 2.5-fold at 24 h of treatment (Fig 4A). Osmotic stress simulated using PEG 6000 showed no significant change in *GhSOS1* expression in the roots before 12 h of treatment, but in the leaves, the expression level increased 2.5-fold in the first 3 h and reached 4.5-fold at 24 h of treatment (Fig 4B). Low temperature stress increased the expression level of *GhSOS1* in both the roots and leaves in the first hour of treatment (approximately 3-fold in the roots and 2.5-fold in the leaves), and subsequently there was a minor decrease in expression after 6 h of treatment (Fig 4C).

### Complementation of a yeast Na^+^/H^+^ antiporter mutant with *GhSOS1*

**Fig 5:**
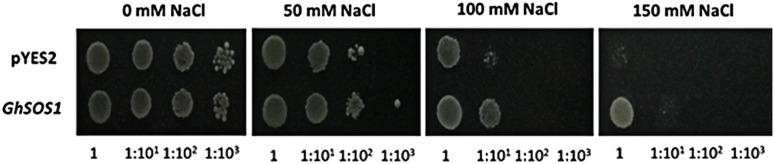
Functional complementation of a salt-sensitive AXT3 yeast cell mutant using *GhSOS1*. AXT3 cells transformed with empty vector (pYES2) or the indicated combination of the *GhSOS1* gene were grown overnight on selective medium. Three microliters of serial dilutions (10^−1^) were spotted onto plates containing SC-U medium (as SC but lacking uracil) supplemented with 0, 50, 100 and 150 mM NaCl. The plates were incubated at 30°C for 2–4 days.

We used the yeast strain AXT3, in which all endogenous sodium transporters were disrupted, to study the function of *GhSOS1*. The yeast expressing *GhSOS1* grew identically to those expressing the empty vector pYES2 under no-saline conditions, but the former grew much better than the latter under 50, 100 and 150 mM NaCl conditions (Fig 5). These results indicated that the GhSOS1 protein played an important role in securing Na^+^ efflux.

### Overexpression of *GhSOS1* improved salinity tolerance in transgenic *Arabidopsis*

**Fig 6:**
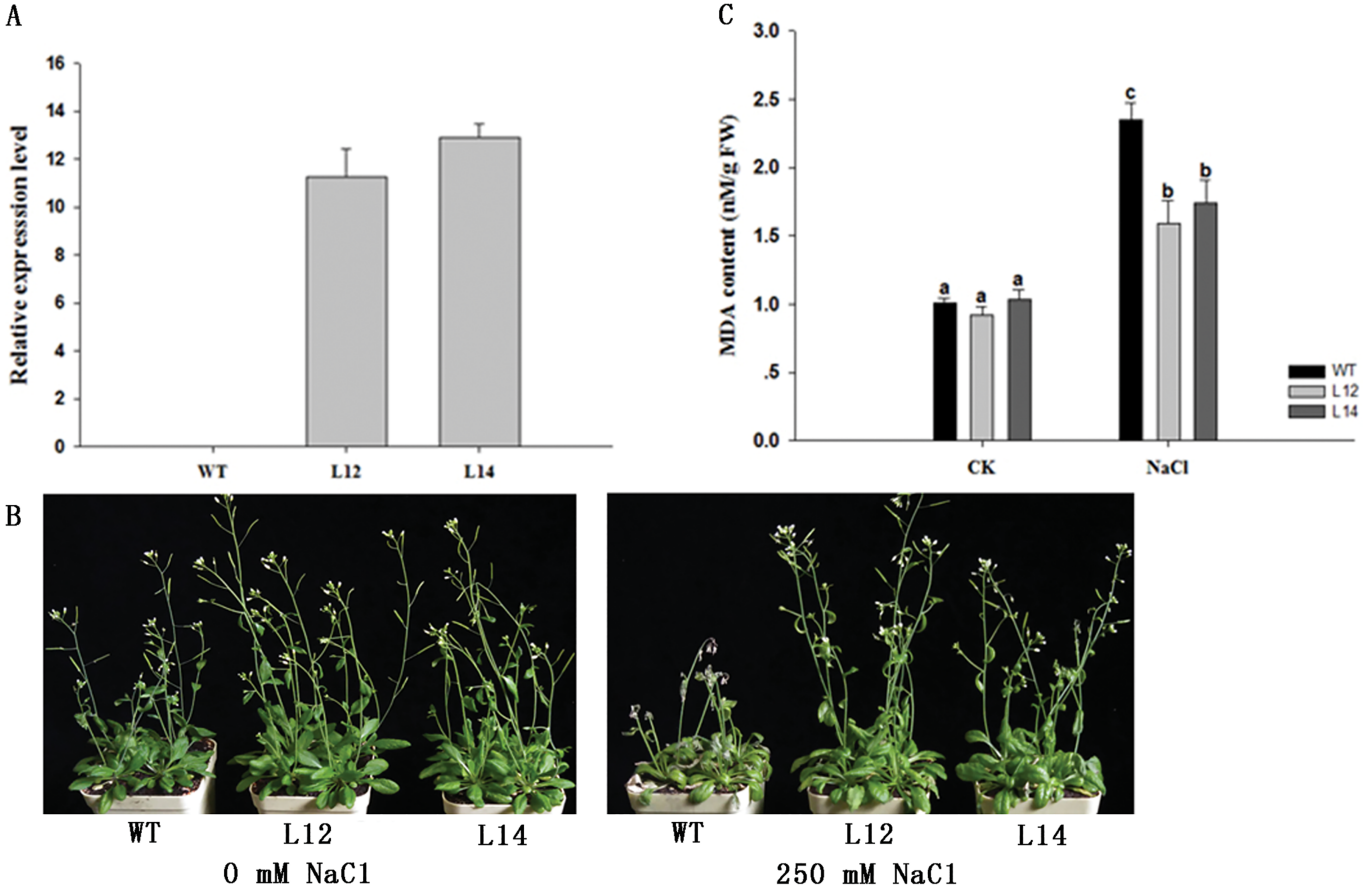
Salt tolerance of *GhSOS1* transgenic *Arabidopsis* plants. (A) *GhSOS1* expression in wild type (WT) and transgenic lines (L12 and L14). (B) Responses of transgenic and WT *Arabidopsis* plants grown in pots under normal conditions and salt stress. (C) MDA content of WT and transgenic plants with or without salt stress treatments. Columns marked with different lower case letters indicate a significant difference (p < 0.05) from the WT treatment.

The expression of *GhSOS1* in transgenic *Arabidopsis* was determined using quantitative RT-PCR analysis (Fig 6A) and two independent T_3_ *GhSOS1* overexpression lines (L12 and L14) were selected to examine salinity resistance. To examine the function of *GhSOS1* in plants, we investigated the tolerance to high salinity of transgenic plants grown at soil watered with NaCl solution. There was no obvious morphological difference between the transgenic lines and wild-type plants under normal conditions, but the growth of transgenic lines was much better than that of wild-type plants after salt treatment for 7 d (Fig 6B). In addition, salt treatment severely affected the growth of wild-type plants, with the flowers and leaf blades exhibiting severe wilting. We measured MDA content in wild- type and transgenic plants because MDA, as an end product of lipid peroxidation, is a reliable indicator of membrane injury under stress conditions [28]. There was no significant difference in the MDA content between wild- type and transgenic lines under normal conditions. However, more MDA accumulated in wild-type plants than in the transgenic lines after salt treatment (Fig 6C).

**Fig 7:**
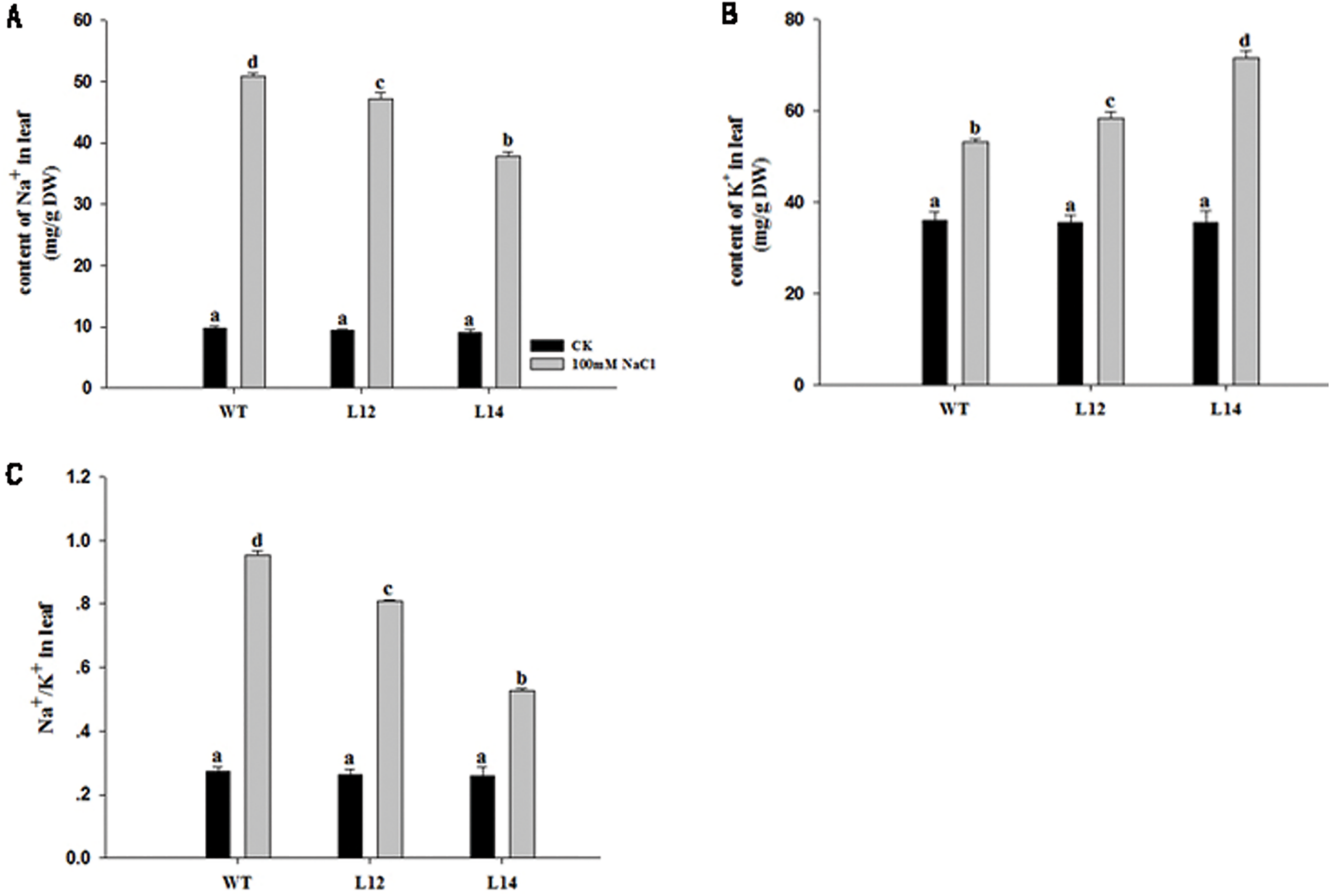
Sodium and potassium content in *GhSOS1*-overexpressing and control plants. (A) Na^+^ content; (B) K^+^ content; (C) Na^+^/K^+^ ration. (WT indicates wild-type *Arabidopsis*). Columns marked with different lower case letters indicate a significant difference (p < 0.05) from the WT treatment.

As SOS1, a plasma membrane Na^+^/H^+^ antiporter that plays a critical role in Na^+^ extrusion and maintaining a low cytosolic Na^+^/K^+^ ratio, is closely associated with plant salt tolerance [29], we measured the Na^+^ and K^+^ content in the leaves. No significant difference in the Na^+^ content, K^+^ content and Na^+^/K^+^ ratio was observed between wild- type and transgenic lines under normal conditions (Fig 7). However, the Na^+^ content was significantly higher in wild- type than in transgenic lines under salt stress, although the Na^+^ content increased in wild- type and transgenic plants under the same conditions. In addition, the K^+^ content decreased in wild-type plants, but dramatically increased in the transgenic lines. Therefore, the transgenic plants displayed a significantly lower Na^+^/K^+^ ratio than the wild-type plants under salt stress (Fig 7). These results indicated that the overexpression of *GhSOS1* in *Arabidopsis* may promote the extrusion of Na^+^ and uptake of K^+^ to maintain a low Na^+^/K^+^ ratio in plant cells and improve the salt tolerance of transgenic plants.

### Expression of salt-stress-related genes was upregulated in the *GhSOS1* transgenic plants

**Fig 8:**
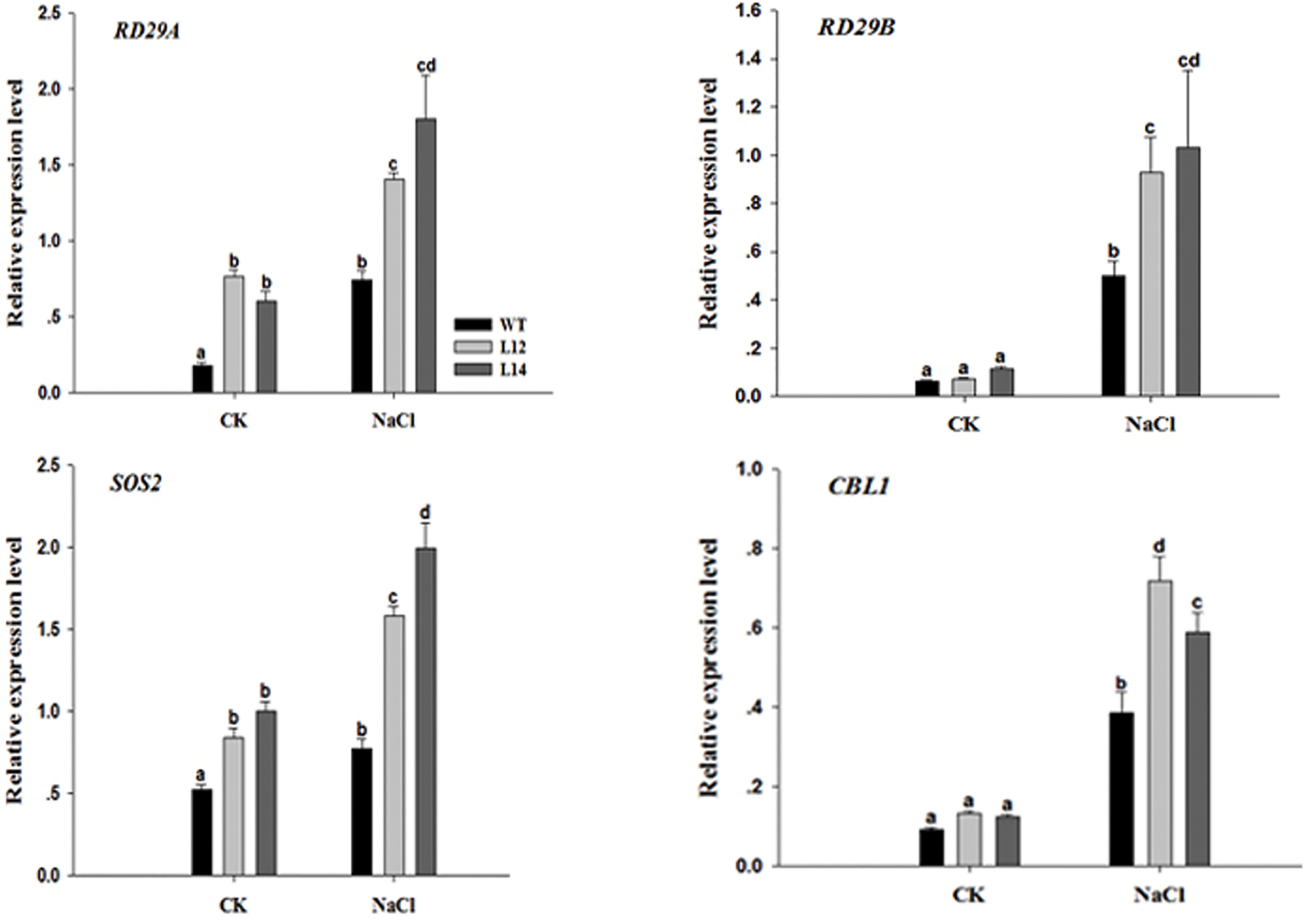
Expression levels of stress-responsive genes (*RD29A*, *RD29B*, *SOS2* and *CBL1*) in control plants and transgenic lines. Columns marked with different lower case letters indicate a significant difference (p < 0.05) from the WT treatment.

Because the overexpression of *GhSOS1* in *Arabidopsis* led to salt tolerance, we selected several salt stress-related genes, including *RD29A*, *RD29B*, *SOS2* and *CBL1*, which act as markers for monitoring salt stress response pathways in *Arabidopsis* and examined the expression levels of these genes using quantitative RT-PCR in wild-type and transgenic plants under both normal and NaCl treatment conditions. The four genes were upregulated in both wild- type and transgenic lines after salt treatment, but the expression levels were dramatically higher in transgenic plants than in wild-type plants (Fig 8). These results suggested that *GhSOS1* may participate in responses to salt stress by regulating the expression of stress-related genes during plant growth and development.

## Discussion

The accumulation of Na^+^ in the cytoplasm is harmful to the growth and development of plants. One mechanism of salt tolerance in plants involves the export of Na^+^ out of the cytoplasm to external medium through plasma membrane Na^+^/H^+^ antiporters. In the present study, we isolated the plasma membrane Na^+^/H^+^ antiporter *GhSOS1* gene from *G*. *hirsutum*. The predicted product of *GhSOS1* contains 12 transmembrane domains, and these domains are important for salt tolerance. The phylogenic analysis revealed a close relationship to the *SOS1* of *Kosteletzkya virginica*. The transient expression analysis of GhSOS1-GFP fusion proteins in onion epidermal cells indicated that GhSOS1 was located at the plasma membrane, consistent with *HtSOS1* [17]. The yeast strain AXT3 was completely complement through the overexpression of *GhSOS1* (Fig 5), showing that GhSOS1 is a Na^+^/H^+^ antiporter. Thus, we concluded that *GhSOS1* encodes a plasma membrane Na^+^/H^+^ antiporter.

The expression level of the *SOS1* gene is upregulated under salt stress conditions [11, 17]. In the present study, there was no obvious change of *GhSOS1* expression level in the roots and leaves under no salt treatment, but its expression was immediately upregulated in roots upon salt stress. Similarly, the expression levels of *ThSOS1* and *SbSOS1* were also higher in the roots than in the shoots [30, 31]. The expression level of *AtSOS1* was stabilized after dehydration in transgenic *Arabidopsis* [32]; however, this effect was not observed in either *GhSOS1* (which was upregulated in the roots and leaves after treated with PEG 6000) or *TaSOS1*[13]. The expression of *AtSOS2* increased in transgenic plants after salt treatment (Fig 8), and *AtSOS2* expression, which is directly induced by salt stress [33], may be regulated through the feedback of *GhSOS1*.

Generally, salt stress initially causes osmotic stress, subsequently followed by physiological drought stress [15]. We examined the physiological function of *GhSOS1* in *Arabidopsis* and observed that the transgenic plants grew much better than WT after salt treatment (Fig 6B). Similarly, the overexpression of *HtSOS1* in rice also improved the salt tolerance of transgenic plants [18]. The production of abundant active oxygen in plant cells leads to oxidative damage to membrane proteins and lipids under salt stress. The peroxidation of membrane lipids generates a great deal of MDA. Therefore, the MDA content is an important physiological trait representing the extent of membrane damage and plant tolerance to salt stress [34]. Analysis of the MDA content revealed that the *GhSOS1* transgenic plants suffered less damage than the control plants under salt stress.

The high Na^+^ concentration in plant cells interferes with the normal metabolism of plants under salt stress [35]. Maintaining the Na^+^/K^+^ balance is vital to maintain the enzyme activity and electric potential of the cell membrane. Under salt stress, reconstruction of the Na^+^/K^+^ balance provides osmotic protection and enables plants to gain salt tolerance [36]. The overexpression of *CcSOS1* in *Chrysanthemum* led to a higher K^+^/Na^+^ ratio in transgenic plants [37]. Our results indicated that *GhSOS1* played an important role in exhausting Na^+^ from the cytoplasm in transgenic plants. As K^+^ is an indispensable essential macronutrient for plant development, this compound is crucial to maintain adequate K^+^ concentrations to prevent plant cells from Na^+^ toxicity [38]. The results of the present study showed that the concentration of K^+^ increased after salt treatment, which may be affected by *GhSOS1*, which influences K^+^ transport via its effect on the H^+^ gradient across the cell membrane. The activity of the K^+^/H^+^ transporter may be coupled with SOS1 through H^+^ cycling. We speculated that the enhanced tolerance to salt stress primarily reflected the increased expression of salt stress-responsive genes: *RD29A*, *RD29B*, *SOS2* and *CBL1*. The expression of these genes was upregulated, and these genes may have cooperated with *GhSOS1* to improve the salt tolerance of transgenic plants.

In summary, the plasma membrane Na^+^/H^+^ antiporter gene *GhSOS1* was cloned from *G*. *hirsutum* and its expression was induced through abiotic stresses. The overexpression of *GhSOS1* enhanced the expression of stress-inducible genes and improved the salt stress tolerance of transgenic *Arabidopsis*. Hence, *GhSOS1* may present a potential target gene for enhancing the salt tolerance of transgenic plants.

## Supporting information

S1 Table Primers for isolation and quantitative RT-PCR (qRT-PCR) analysis. Gh: cotton, At: *A*. *thaliana*. *GhActin* is the cotton gene encoding actin, and *AtActin* is the *A*. *thaliana* gene encoding actin.
(DOCX)
