# Supplementary material for: GhSOS1, a plasma membrane Na+/H+ antiporter gene from upland cotton, enhances salt tolerance in transgenic Arabidopsis thaliana
Source: PLoS One. 2017 Jul 19;12(7):e0181450. doi: 10.1371/journal.pone.0181450 (PMC5517032; doi:10.1371/journal.pone.0181450)
Supplement: S1 Table — Gh: cotton, At: A. thaliana. GhActin is the cotton gene encoding actin, and AtActin is the A. thaliana gene encoding actin. (DOCX) [file pone.0181450.s001.docx]

**Supplementary materials**

**S1 Table.** Primers for isolation and quantitative RT-PCR (qRT-PCR) analysis.

Gh: cotton, At: *A. thaliana*. *GhActin* is the cotton gene encoding actin, and *AtActin* is the *A. thaliana* gene encoding actin.

| Gene Names | Sense primers (5′-3′) | Antisense primers (5′-3′) | Function |
| --- | --- | --- | --- |
| *GhSOS1* | GCTCTAGAATGGAGGAAGTGAAAGAGTATC | ACGCGTCGACTTAAGAAGCCTGGTGGAATG | cDNA isolation |
| *GhSOS1* | TGGGAAGGATTGGTGATGGC | GGACCAGCAAGCAGAACCAT | Real time PCR |
| *GhSOS1* | CACGGGGGACTCTAGAATGGAGGAAGTGAAAGAGT | ACTCATACTAGTCCCGGGAGAAGCCTGGTGGAATGATA | recombinant plasmid construct for  Subcellular localization |
| *GhSOS1* | GGACTCTAGAGGATCCATGGAGGAAGTGAAAGAGT | GATCGGGGAAATTCGAGCTCTTAAGAAGCCTGGTGGAAT | recombinant plasmid construct for  overexpression |
| *GhSOS1* | CGTTACTAGTGGATCCATGGAGGAAGTGAAAGAG | AGGGAATATTAAGCTTTTAAGAAGCCTGGTGGAA | recombinant plasmid construct for  complementation in yeast |
| *GhActin* | ATCCTCCGTCTTGACCTTG | TGTCCGTCAGGCAACTCAT | Real time PCR |
| *AtActin* | GAAATCACAGCACTTGCACC | AAGCCTTTGATCTTGAGAGC | Real time PCR |
| *AtRD29A* | TGAAAGGAGGAGGAGGAATGGTTGG | ACAAAACACACATAAACATCCAAAGT | Real time PCR |
| *AtRD29B* | CCAGATAGCGGAGGGGAAAGGACAT | AAGTTCACAAACAGAGGCATCATCATCATAC | Real time PCR |
| *AtSOS2* | GGCTTGAAGAAAGTGAGTCTCG | GCTACATAGTTCGGAGTTCCACA | Real time PCR |
| *AtCBL1* | GAAATGAAACTGGCTGATGAAACCATAGAG | CTCGTGGCAATCTACTCGGTCTTAAACC | Real time PCR |
